# The Relationship of Compressive Strength and Chemically Bound Water Content of High-Volume Fly Ash-Cement Mortar

**DOI:** 10.3390/ma14216273

**Published:** 2021-10-21

**Authors:** Chunping Gu, Jikai Yao, Yang Yang, Jie Huang, Linhao Ma, Tongyuan Ni, Jintao Liu

**Affiliations:** 1 College of Civil Engineering, Zhejiang University of Technology, Hangzhou 310023, China; guchunping@zjut.eud.cn (C.G.); 2111906021@zjut.edu.cn (J.Y.); 2111706008@zjut.edu.cn (J.H.); 2111706028@zjut.edu.cn (L.M.); hznity@zjut.edu.cn (T.N.); jtliu@zjut.edu.cn (J.L.); 2 Key Laboratory of Civil Engineering Structures & Disaster Prevention and Mitigation Technology of Zhejiang Province, Zhejiang University of Technology, Hangzhou 310023, China; 3 Zhejiang Construction Investment Group Co., Ltd., Hangzhou 310013, China

**Keywords:** high-volume fly ash-cement-based material, compressive strength, chemically bound water content

## Abstract

Fly ash (FA) has been widely used in cement-based materials, but limited work has been conducted to establish the relationship between the compressive strength and hydration process of high-volume FA (HVFA)-cement-based material. In this study, the compressive strength and chemically bound water contents of FA-cement-based materials with different water-to-binder ratios (0.4, 0.5, and 0.6) and FA contents (0%, 30%, 40%, 50%, 60%, and 70%) were tested. Replacing more cement with FA reduced the compressive strength and of HVFA-cement-based materials. The compressive strength and chemically bound water content reduced by about 60–70% when 70% cement was replaced by FA. Water-to-binder ratio showed more significant influence on the chemically bonded water at later ages than that at early ages. Based on test results, the prediction equation of chemically bound water content was established, and its accuracy was verified. The error was less than 10%. The relationship between the compressive strength and chemically bound water content was also fitted. The compressive strength and chemically bound water content showed linear relationships for different water-to-binder ratios, hence the compressive strength of HVFA-cement mortar could be predicted with the chemically bound water content and water-to-binder ratios. The results of this study could be used for the prediction of the compressive strength development of HVFA-cement mortars, and is helpful to develop the mix design method of HVFA-cement-based materials.

## 1. Introduction

The number of coal-fired electricity plants in China is always increasing, which results in the increasing output of fly ash (FA). Initially, FA was recognized as an environmental pollutant, so numerous studies have been carried out on FA treatment and disposal [1]. FA can be recycled for various applications, such as soil amelioration, the construction industry, the ceramic industry, catalysis, depth separation, zeolite synthesis, and valuable metal recovery [2,3]. In the construction industry, FA was mainly used as a raw material for cement production and as a substitute for cement in construction materials [4,5,6,7,8]. In the last decade, FA-based alkali-activated binders have emerged as a promising new cement alternative in the field of building and construction materials [9,10,11,12,13,14]. The incorporation of FA not only reduces the usage of cement, but also improves the performance and quality of cement-based materials [15,16,17,18,19,20,21], and thus the applications of FA in cement-based materials have been very extensive.

Existing studies show that FA exhibits the pozzolanic effect, morphological effect, and micro-aggregate effect in cement-based materials [22,23]. The pozzolanic effect of FA could improve the durability and late age mechanical properties of cement-based materials [7,24,25,26,27]. However, the pozzolanic relativity of FA is quite low, so the replacement of cement with FA will reduce the early-age strength of cement-based materials [28]. Due to the morphological effect, the workability of cement-based materials could be improved [19,26,29], and the autogenous and drying shrinkage of cement-based materials could be restricted due to the micro-aggregate effect of FA [30,31].

In addition, the existence of FA also affects the hydration process of cement-based materials [25,32,33,34]. FA shows a much lower reactivity than cement. At early ages, replacing cement with FA would reduce the total hydration degree of a FA-cement binary system, but promote the hydration degree of cement [35,36]. Due to the pozzolanic reactivity of FA, the hydration degree of FA would continuously increase at late ages, so the final hydration degree of the FA-cement binary system could be even higher than pure cement system under the same circumstances [37].

The replacement of cement with FA could reduce the early-age hydration heat and the shrinkage of concrete, so FA concrete, especially high-volume FA (HVFA) concrete, has been widely used in mass concrete structures [16,32,38]. The FA content in the binder of mass concrete could be as high as 80% in some special mass concretes [39]. Compared with normal FA concrete, i.e., concrete with a relatively low content of FA, HVFA concrete contains a lower content of cement. Hence its hydration process and performance development are different from that of normal FA concrete [28,37,40]. With the increase of FA content, the early age strength of concrete is lower, and longer time is required to reach its final strength [41]. Meanwhile, with the increase of FA content, the pH value in the pore solution of cement-based materials decreases, and the reaction degree of FA decreases [42]. Due to the special characteristics of HVFA concrete, the mix design methods for normal concrete are not applicable to HVFA concrete [39,43]. However, the proper mix design method for HVFA concrete is crucial for its wider application. Normally, the mix design of concrete is based on the strength requirements. However, the compressive strength prediction method for HVFA-cement-based materials has not been well established. The strength development of HVFA-cement-based materials is mainly determined by its hydration process. Hence it is possible to develop the mix design method for HVFA concrete based on the relationship between the strength and hydration degree of HVFA-cement binary system. The strength development and hydration process of FA-cement system have been extensively studied, but less attention was focused on the relationship between them, especially for HVFA-cement-based materials.

In this paper, the compressive strength of HVFA-cement mortars with different water-to-binder ratios (*w/b*) (0.4, 0.5 and 0.6) and FA contents (0%, 30%, 40%, 50%, 60%, and 70%) were tested at the different ages, and the chemically bound water contents, which indicates the hydration degree, were determined at different ages with ignition method. According to the test results, the chemically bound water content was related to *w/b*, FA content, and curing ages, and the prediction formula for chemically bound water content was proposed and validated. Furthermore, the relationship between the compressive strength of HVFA mortar and the chemically bound water content was fitted. It is helpful to predict strength development and develop a mix design method of HVFA-cement-based materials.

## 2. Materials and Methods

### 2.1. Research Program

**Figure 1:**
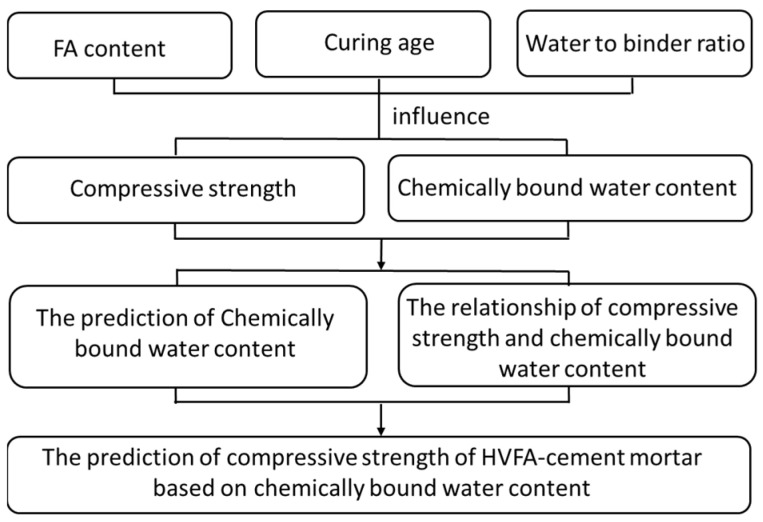
The flowchart of this study.

The flowchart of this study is shown in Figure 1. Generally, this study was performed following three steps:

Step 1: Reveal the effect of FA content, water-to-binder ratio, and curing age on the compressive strength and chemically bound water contents of HVFA-cement mortars with experimental studies;
Step 2: Propose and verify the prediction formula for the chemically bound water content of HVFA-cement mortars based on the FA content, water-to-binder ratio, and curing age, according to the test results;
Step 3: Establish the relationship between the compressive strength and chemically bound water content, then the compressive strength of HVFA-cement mortars could be predicted based on the chemically bound water content.

### 2.2. Materials and Mix Proportions

The raw materials used in this study included cement, FA, sand, and superplasticizer. The cement was P·I 42.5 Portland cement in accordance with GB 175-2007 [44], with a specific surface area of 342 m^2^/kg and an apparent density of 3.16 g/cm^3^. FA is a type of class II FA in accordance with GB/T 1596-2017 [45], and its water demand ratio was 98.8%. The main chemical compositions of the cement and FA are shown in Table 1. The sand was ISO standard sand in accordance with ASTM C778-17 [46]. Subote PCA^®^-300P polycarboxylic acid superplasticizer was added to control all the mortar’s fluidity at 280 ± 20 mm. The fluidity tests were performed according to GB 2419-2005 [47]. After mixing, the fresh mortar was poured into a conical mold on a flow table. The top and bottom diameters of the conical mold were 70 mm and 100 mm, respectively. The height of the conical mold was 60 mm. After the conical mold was removed, the flow table was vibrated 25 times in 25 s. Then the diameters of the mortar in two directions perpendicular to each other were measured, and the average value was used as the fluidity of the cement mortar.

The mix proportions of HVFA-cement mortars are shown in Table 2. The mix proportions of HVFA-cement mortars were selected based on ISO 679-2009 [48], in which a batch of mortar specimens consists of 450 g cement, 1350 g sand, and 225 g water. In particular, the FA contents of HVFA-cement mortar or HVFA concrete is in the range of 30–70%, and the water-to-binder ratio is normally between 0.4 and 0.6. Therefore, the mix proportions of HVFA-cement mortars as shown in Table 2 were selected. FA was used to replace part of the cement.

### 2.3. Compressive Strength Tests

The compressive strength tests were performed in accordance with ISO 679-2009 [48]. The size of the specimens was 40 mm × 40 mm × 40 mm. The specimens were demolded at 24 h, then cured in standard curing room (20 ± 2 °C, RH > 95%). The compressive strength of the mortars was tested at the ages of 3 d, 7 d, 28 d, 60 d, and 90 d. Six specimens were used for each batch.

### 2.4. Chemically Bound Water Content Test

The ignition method was applied to test the chemically bound water content of HVFA-cement pastes [42]. The fresh HVFA-cement pastes were cast into the molds with a size of 40 mm × 40 mm × 40 mm. The specimens were also demolded at 24 h, and then cured in a standard curing room. The curing ages were set to be 3 d, 7 d, 28 d, 60 d, and 90 d, which were the same to FA-cement mortar specimens. After curing, the specimens were crushed into small pieces, then the pieces in the center of the specimen were immersed in ethanol to stop the hydration. Before the chemically bound water content test, the samples were ground into powders. Then the powders were placed in an oven at 105 °C for 24 h until the mass of powders became the constant. After that, the dried powders were placed in a muffle furnace at 950 °C for 3 h to remove the chemically bounded water.

The chemically bound water content (*w_cbw_*, %) was determined with Equation (1) [49].

(1) $$w_{cbw}=\frac{\frac{m_{1}-m_{2}}{m_{1}}-\alpha_{b}}{1-\alpha_{b}}$$

where *m*_1_ (g) is the mass of the powder samples after drying at 105 °C; *m*_2_ (g) is the mass of the powder samples after drying at 950 °C; *α_b_* (%) is the ignition loss of the binders, which can be calculated with Equation (2).

(2) $$\alpha_{b}=L_{FA}\cdot \alpha_{FA}+L_{c}\cdot \alpha_{c}$$

where *L_FA_* (%) and *L_c_* (%) are the mass fraction of FA and cement in the binder respectively; *α_FA_* (%) and *α_c_* (%) are the ignition loss of FA and cement, respectively. The average value of three samples were used as the final result.

## 3. Results and Discussions

### 3.1. Compressive Strength of HVFA-Cement Mortars

**Figure 2:**
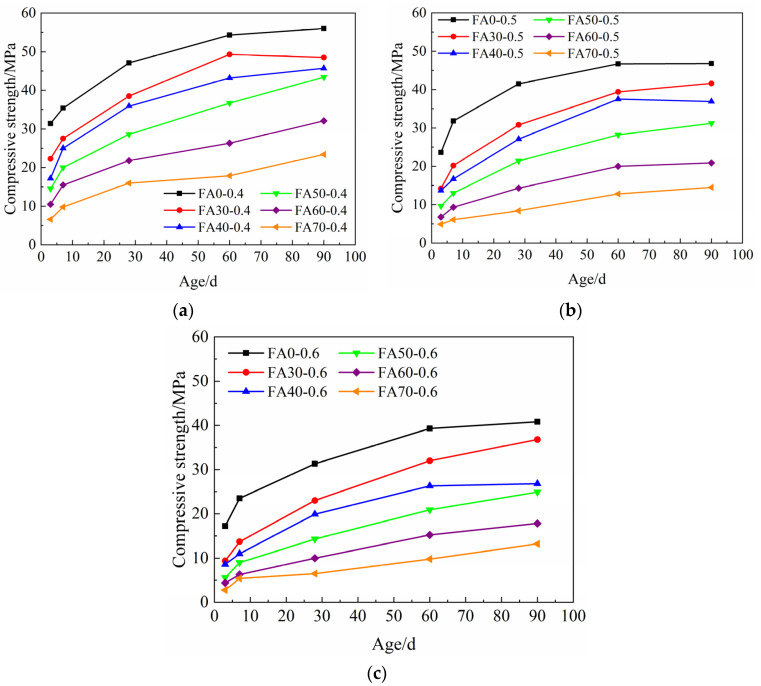
Compressive strength of HVFA-cement mortars at different ages: (**a**) *w/b* = 0.4; (**b**) *w/b* = 0.5; (**c**) *w/b* = 0.6.

The compressive strength developments of HVFA-cement mortars are shown in Figure 2. It can be seen that:

(1). The compressive strength of the mortars improved along with the curing age, and the compressive strength grew fast at early ages and relatively slowly at late ages. When the FA content grew higher, the compressive strength development of mortars at early ages was slower, but the growth of compressive strength at late ages was generally faster. In other words, with the increase of FA content, the growth rate of the compressive strength of mortars decreased at early ages, but increased at late ages. For example, from 3 d to 7 d, the compressive strengths of FA0-0.5 and FA70-0.5 increased by 8.2 MPa and 1.2 MPa respectively, while from 60 d to 90 d, the compressive strengths of FA0-0.5 and FA70-0.5 increased by 0.1 MPa and 1.7 MPa, respectively. The pozzolanic reactivity of FA contributed to the long-term strength improvement of the HVFA-cement mortar.
(2). With the increase of FA content, the compressive strength of HVFA-cement mortar decreased. When the FA content was low (e.g., 30%), the compressive strength of HVFA-cement mortar at 90 d can be close to that of pure cement mortar. For instance, the compressive strength of FA30-0.6 at 90 d was 90.2% of the compressive strength of FA0-0.6, and for FA30-0.4 and FA30-0.5, their 90 d compressive strengths were 86.6% and 88.9% of the pure cement mortars under the same circumstances. When the FA content was high (i.e., >50%), the compressive strength of HVFA-cement mortar decreased remarkably. The compressive strengths of FA70-0.4, FA70-0.5 and FA70-0.6 at 90 d was only 41.8%, 31.0% and 32.4% of the corresponding pure cement mortars. This was mainly because the reactivity of FA was much lower than cement. With the increase of cement replaced by FA, the actual water-to-cement ratio in the paste increased, and the overall reaction degree decreased, resulting in the loose microstructure of the paste and the decrease of the compressive strength [49].

**Figure 3:**
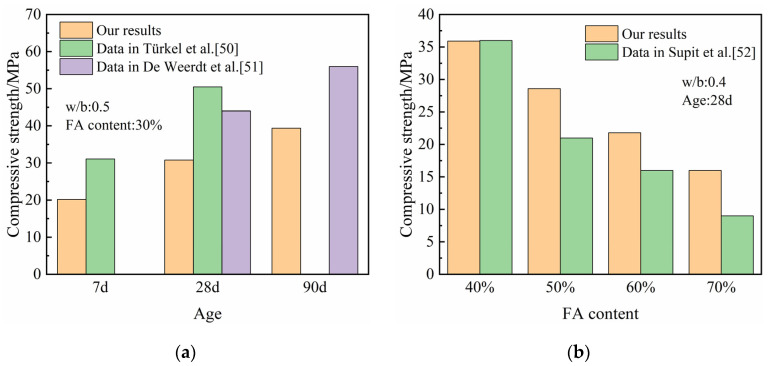
The comparison of compressive strength of HVFA-cement mortars in this study and existing literature [50,51,52]. (**a**) *w/b*: 0.5, FA content: 30%; (**b**) *w/b*: 0.4, Age 28 d.

The comparisons of the compressive strength results in this study and existing literature on the compressive strength of HVFA-cement mortar are shown in Figure 3. It can be seen that, generally, the compressive strength HVFA-cement mortars in the literatures [50,51,52] also increased with curing age and decreased with the increase of FA content. The differences in the compressive strengths under the same FA content, water-to-binder ratio and curing age are resulted from the difference in the properties of the raw materials in these studies.

Compared with other widely used supplementary cementitious materials, for example silica fume (SF) and blast furnace slag (BFS), FA caused more compressive strength reduction. When less than 50% cement was replaced with BFS, the concrete could exhibit comparable compressive strength to the plain concrete [53,54]. If excess BFS was added, the compressive strength will also reduce. SF has very high pozzolanic reactivity, the addition of SF (<15%) could remarkably improve the compressive strength of cement-based materials. However, more SF may also cause compressive strength reduction due to the aggregation of SF particles [55].

**Figure 4:**
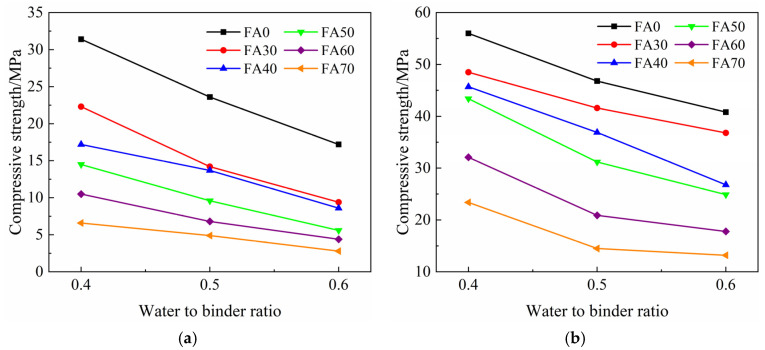
Relationship between the compressive strength and *w/b* of mortars: (**a**) 3 d; (**b**) 90 d.

Figure 4 shows the influence of *w/b* on the compressive strength of HVFA-cement mortars at 3 d and 90 d. It can be seen that the 3 d compressive strength of HVFA-cement mortar decreased linearly with the increase of *w/b* and the influence of *w/b* was less significant when the FA content was higher. In other words, at early ages, the effect of FA content on the compressive strength was more remarkable when *w/b* was lower. In cement-based materials, besides the pozzolanic effect, FA also exhibited the micro-aggregate effect [22]. When *w/b* was lower, the microstructure of the mortar was denser, and thus the micro-aggregate effect of FA would be enhanced. Therefore, FA had a great impact on the compressive strength of FA mortar when *w/b* was lower. At late ages, e.g., 90 d, the compressive strength of HVFA-cement mortars also decreased with the increase of *w/b*. The pozzolanic reaction of FA has taken effect at 90 d, and the microstructure of HVFA mortars became dense. Hence compared with that at early ages, the effects of the FA content on the late age compressive strength became more significant at a high *w/b*, i.e., at 0.6.

### 3.2. Chemically Bound Water Content of HVFA-Cement Mortars

**Figure 5:**
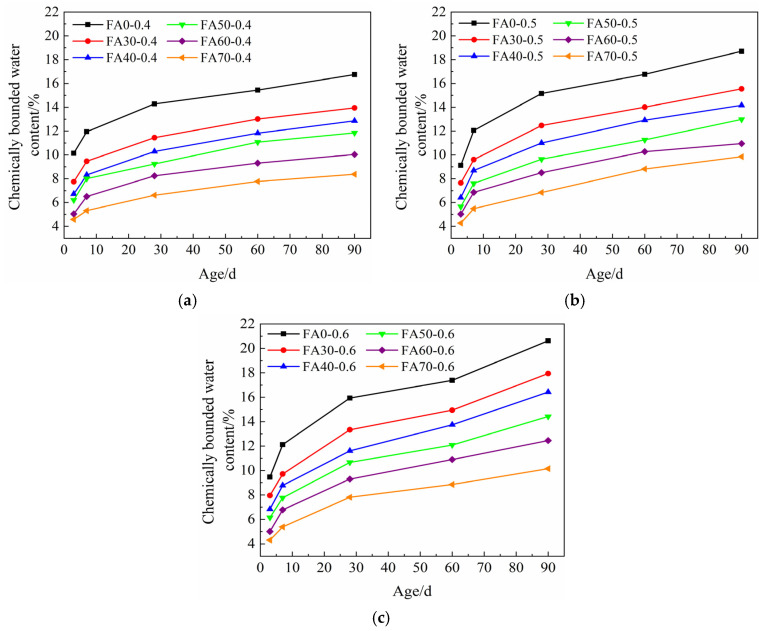
Chemically bound water contents of FA-cement pastes with different *w/b* at different curing ages: (**a**) *w/b* = 0.4; (**b**) *w/b* = 0.5; (**c**) *w/b* = 0.6.

The evolution of the chemically bound water content of HVFA-cement pastes with different *w/b* along with the curing age is shown in Figure 5. It can be seen that in accordance with the compressive strength development of HVFA-cement mortars, the chemically bound water content of HVFA-cement paste also increased along with the curing age, with a fast growth rate at early ages and a slow growth rate at late ages. As the hydration reaction preceded, the amount of free water in the paste was continuously decreasing, and the microstructure of the paste was becoming denser, which led to the strength development.

The FA content had a great influence on the chemically bound water content of HVFA-cement pastes. With the increase of FA content, the chemically bound water content of pastes decreased. At the age of 90 d, although the pozzolanic reaction was still ongoing, there was a large gap between the chemically bound water contents of HVFA-cement pastes and the pure cement paste. As shown in Figure 5, the chemically bound water contents of FA70 at different *w/b* were only about 40% of pure cement paste. The reasons are two-fold: 1. the reactivity of FA is much lower than that of cement, so the overall reaction rate and early reaction degree of the pastes were lower when FA was added [56]; 2. the lower amount of Ca(OH)_2_ in HVFA-cement paste inhibited the pozzolanic reaction of FA [57]. The classic chemical reactions of FA blended cement can be described as follows [58,59]:

(3) $$C_{3}S+5.3H\to C_{1.7}{\mathrm{SH}}_{4}+1.3\mathrm{CH}$$

(4) $$C_{2}S+4.3H\to C_{1.7}{\mathrm{SH}}_{4}+0.3\mathrm{CH}$$

(5) $$C_{3}A+C\bar{S}H_{2}+10H\to C_{4}A\bar{S}H_{12}$$

(6) $$C_{4}\mathrm{AF}+C\bar{S}H_{2}+14H\to C_{4}A\bar{S}H_{12}{+\mathrm{CH}+\mathrm{FH}}_{3}$$

(7) $$C_{3}A+6H\to C_{3}{\mathrm{AH}}_{6}$$

(8) $$C_{4}\mathrm{AF}+10H\to C_{3}{\mathrm{AH}}_{6}{+\mathrm{CH}+\mathrm{FH}}_{3}$$

(9) $$1.1\mathrm{CH}+S+2.8H\to C_{1.1}{\mathrm{SH}}_{3.9}$$

where C, S, H, A, $\bar{s}$ and F are the abbreviations for CaO, SiO_2_, H_2_O, Al_2_O_3_, SO_3_, and Fe_2_O_3_, respectively. The cement clinker is composed of tricalcium silicate (C_3_S), dicalcium silicate (C_2_S), tricalcium aluminate (C_3_A), and tetracalcium aluminoferrite (C_4_AF). When water was added, C_3_S and C_2_S would react with water to form calcium silicate hydrate (C-S-H) and Ca(OH)_2_(CH). Hence, when cement was partly replaced by FA, the amount of C-S-H and CH would be reduced in the FA-cement paste. Regarding the pozzolanic reactions, FA normally contains amorphous silicate, which would react with CH to form C-S-H with a lower Ca/Si ratio (as shown in Equations (3)–(9)). The pozzolanic reaction of FA requires the consumption of CH. The more FA meant less cement and thus a lower amount of CH. The amount of CH in HVFA-cement paste was lower, which reduced the reaction degree of FA, as well as the overall chemically bound water content [60].

**Figure 6:**
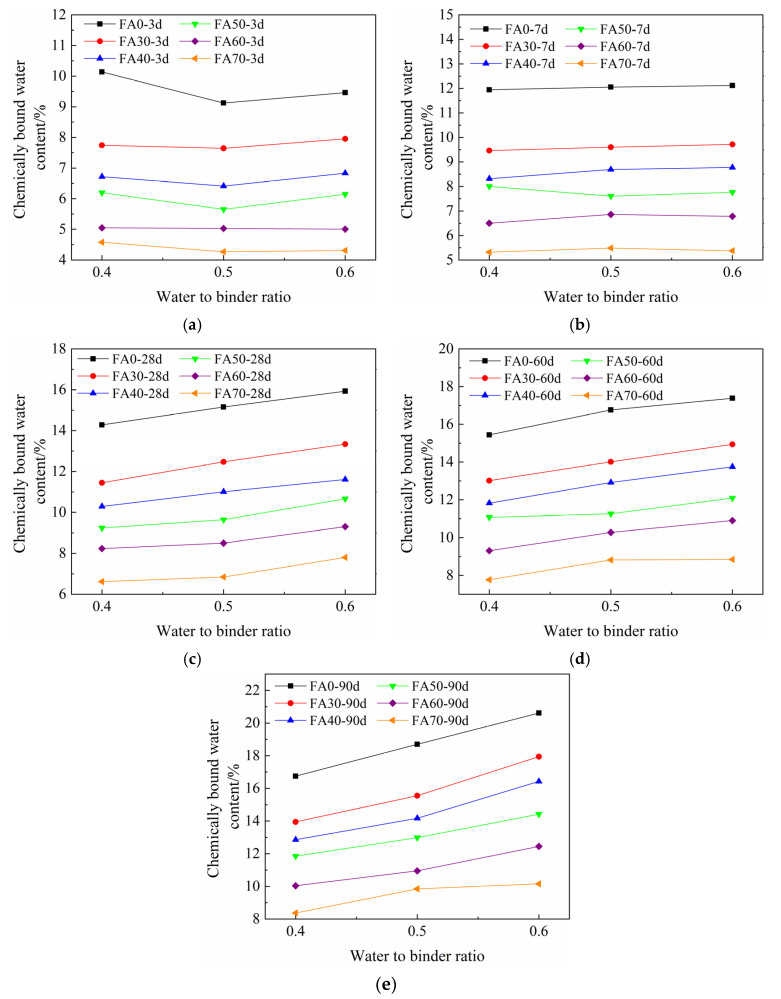
Relationship between the chemically bound water content of pastes and water-binder ratio at different ages: (**a**) 3 d; (**b**) 7 d; (**c**) 28 d; (**d**) 60 d; (**e**) 90 d.

Figure 6 shows the influence of *w/b* on the chemically bound water content of HVFA-cement pastes. In general, at the ages of 3 d and 7 d, *w/b* slightly influenced the chemically bound water content of FA pastes. The chemically bound water content increased with the increase of *w/b*, but the difference was not significant. However, at late ages, e.g., 28 d, 60 d, and 90 d, the chemically bound water content obviously increased with the increase of *w/b*. At early ages, due to the low reactivity of FA, the chemically bound water content of HVFA-cement paste mainly depends on the hydration degree of cement [60]. In this study, the actual water-to-cement ratios of the pastes were relatively high (0.4–2.0), which implied sufficient water supply for the cement hydration. The cement particles in the pastes could fully contact with water, and the excess water did not significantly accelerate the early age hydration of cement. Therefore, the overall hydration rate and degree of the pastes were marginally affected by *w/b*. At late ages, the hydration degree of cement became higher, and the pozzolanic reaction of FA also took place. Hence, the more water in the HVFA-cement paste resulted in higher overall reaction degree in late age paste.

### 3.3. Prediction of Chemically Bound Water Content Based on Curing Age, w/b and FA Content

**Figure 7:**
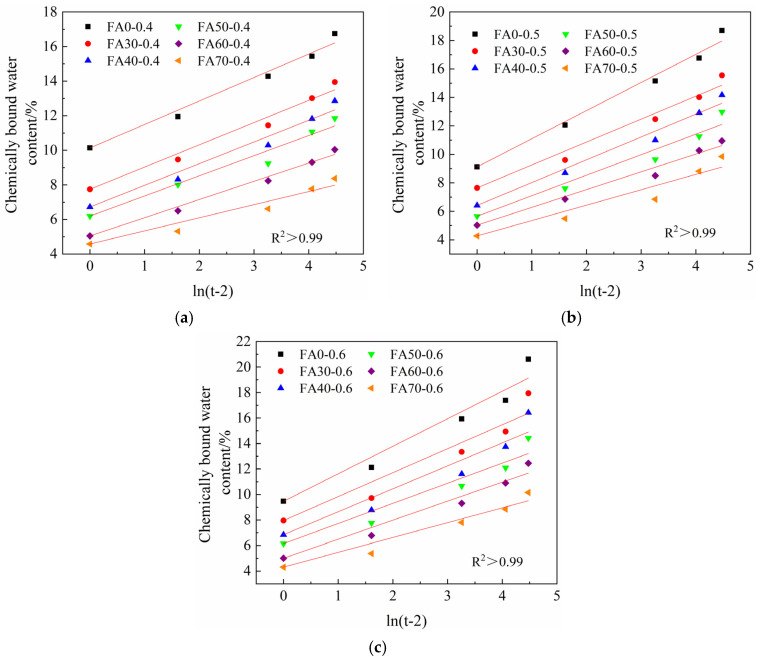
Relationship between the chemically bound water content and logarithm of the curing age: (**a**) *w/b* = 0.4; (**b**) *w/b* = 0.5; (**c**) *w/b* = 0.6.

Under the condition of standard curing, the compressive strength of concrete is proportional to the logarithm of the curing age [42]. The evolution trend of the chemically bound water content of HVFA-cement paste with curing age is similar to that of compressive strength as shown Figure 2 and Figure 5. To quantitatively predict the development of chemically bound water content, this study attempted to use logarithm functions to describe the development of chemically bound water content of HVFA-cement paste. It can be seen from Figure 7 that the relationship between the chemically bound water content of HVFA-cement paste and the logarithm of curing age was basically linear, and can be expressed as:

(10) $$w_{cbw}=a+b\cdot \ln(t-2)$$

where *w_cbw_* is the chemically bound water content, *t* (d) is curing age (*t* ≥ 3), *a* is the measured value of chemically bound water content of HVFA-cement paste at the age of 3 d, *b* is the regression parameter. The correlation coefficients R^2^ were all above 0.99, so this formula can be used to quantitatively characterize the chemically bound water content of HVFA-cement paste. Nevertheless, this formula is only applicable for the prediction of chemically bound water content after the age of 3 d. When the age is less than 3 d, the hydration development of cement-based materials is relatively rapid and complex [61], and the logarithmic function cannot well describe the hydration development process at this age.

Equation (10) only shows the relationship between the chemically bound water content and the curing age. The influences of *w/b* and FA content on the chemically bound water content were introduced by fitting the *b* value with the FA content and *w/b*. According to the test results shown in Figure 5 and Figure 6, a binary linear formula was adopted to fit this relationship, which is:

(11) $$b=0.57-0.01\cdot L_{x}+2.66\cdot w/b$$

where *L_x_* (%) is the FA content, *w/b* is the water-to-binder ratio. The correlation coefficient R^2^ was 0.91, implying a good regression result.

The formula that could predict the chemically bound water content of HVFA-cement paste based on curing age, *w/b* and FA content can be obtained by substituting Equation (11) into Equation (10):

(12) $$w_{cbw}=a+(0.57-0.01\cdot \alpha_{x}+2.66\cdot w/b)\cdot \ln(t-2)$$

**Figure 8:**
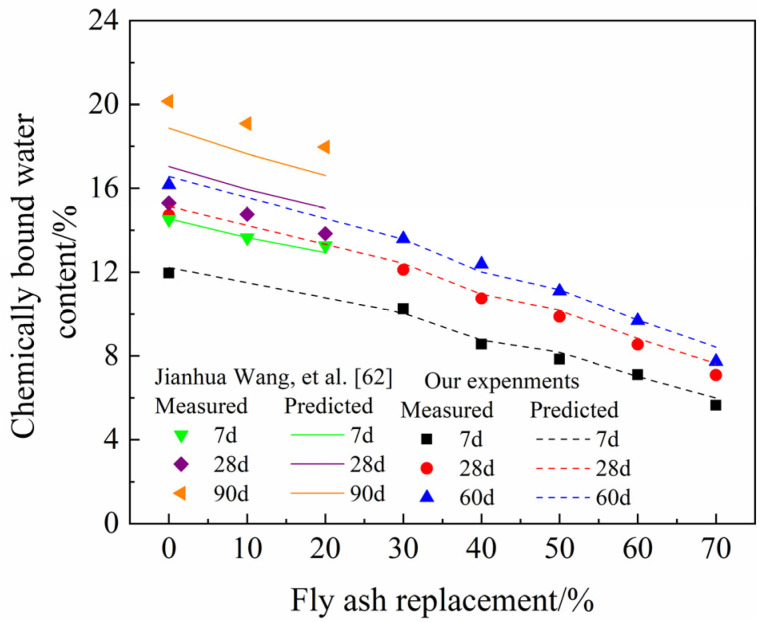
Comparison of the measured and predicted values of chemically bonded water content.

**Figure 9:**
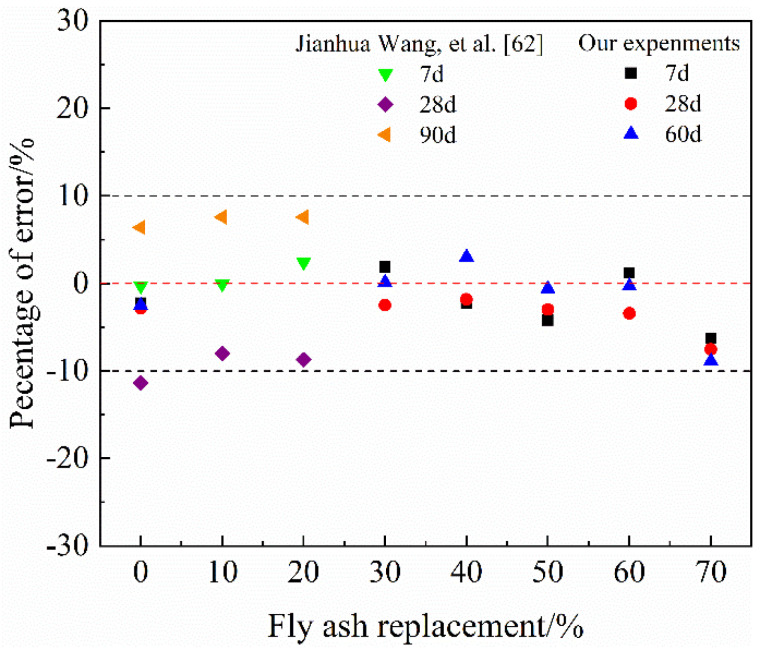
Percentage of the errors.

To verify the accuracy of Equation (12), the predicted results are compared with the experimental results. The experimental results included our own tests results on the chemically bound water content of HVFA-cement pastes with *w/b* at 0.45 and the experimental data on the chemically bound water content of FA-cement paste with *w/b* at 0.35 measured by Wang et al. [62]. The test methods for HVFA-cement pastes with *w/b* at 0.45 were the same as that used in this study. The comparison and the error analysis results are shown in Figure 8 and Figure 9, respectively. It can be seen that Equation (12) could predict the chemically bound water content of HVFA-cement pastes with a high accuracy. As shown in Figure 9, generally, the errors were within 10%, which is acceptable for cement-based materials [63].

### 3.4. The Relationship between the Compressive Strength and Chemically Bound Water Content

**Figure 10:**
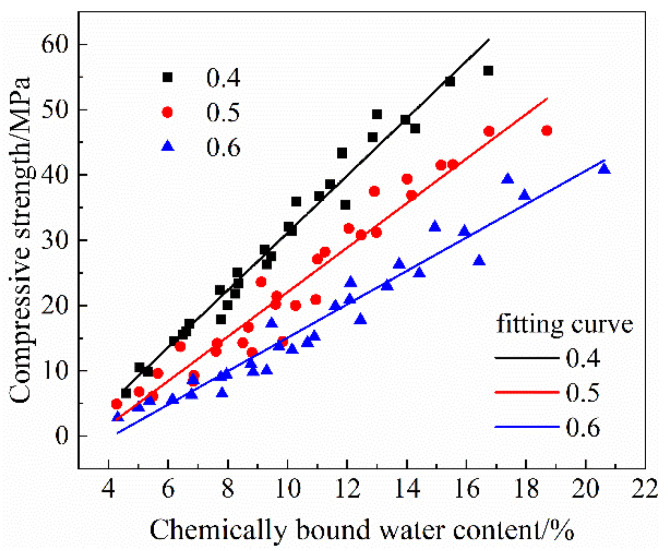
Relationship between the compressive strength and chemically bound water content.

The strength development of cement-based materials is directly related to its hydration process [64]. Figure 10 shows the relationship between the compressive strength and the chemically bound water contents of the cement-based materials. It can be seen that the relationships were approximately linear for different *w/b*. So linear fitting was performed to establish the quantitative relationship between the compressive strength and the chemically bound water content. The results are shown in Table 3. The compressive strength of HVFA-cement mortar can be predicted with Equation (13) when *w/b* was in the range from 0.4 to 0.6.

(13) $$\sigma =k\cdot w_{cbw}+s$$

where *σ* (MPa) is the compressive strength; *w_cbw_* (%) is the chemically bonded water content; *k* is the slope; *s* is the intersection with *y*-axis, and thus, the intersection with *x*-axis is *–s/k*.

As shown in Table 3, the lower the *w/b*, the higher the slope of fitting line. The unit increase of chemically bound water content would lead to higher compressive strength improvement for HVFA-cement mortars with lower *w/b*. When *w/b* was lower, the microstructure of the paste was denser, so the strength developed more rapidly with the ongoing hydration. However, when *w/b* was higher, the microstructure of the paste was looser, and the strength development caused by hydration was slower. In addition, it can be seen from Figure 10 that under the same total chemically bound water content, the smaller *w/b* led to the higher compressive strength. Therefore, to achieve the same compressive strength, the mortar with a higher *w/b* needs a higher total hydration degree.

It also can be seen from Table 3 that the fitting lines intersected the *x*-axis (the chemically bound water content) at 2.90%, 3.52% and 4.11% for *w/b* of 0.4, 0.5, and 0.6, respectively. These values represent the minimum chemically bound water content required for the mortars to sustain the load. When the total hydration degree was higher than the minimum value, the mortars began to exhibit strength. It can also be seen that this value increased with the increase of *w/b*. As *w/b* increased, the amount of cementitious material in unit volume decreased, so a higher hydration degree was required to form a skeleton structure in the paste, and to obtain the ability to resist external forces [65].

It was also quite interesting that both the slope and the *x*-axis intersection were in a linear relationship with *w/b*. The fitted relationships are shown as Equations (14) and (15). The correlation coefficients R^2^ were both above 0.99.

*k* = −9.1 *w/b* + 8.0 (14)

*–s/k* = 6.1 *w/b* + 0.5 (15)

To sum up, based on Equations (12)–(15), the compressive strength of the HVFA-cement mortar could be predicted with *w/b*, FA content, and curing age. So once the requirements on the compressive strength are known, the FA content and water-to-binder ratio of the HVFA-cement-based materials could be determined. This is helpful for the mix design of HVFA-cement-based materials in practice.

The test results on the relationship between the compressive strength and chemically bounded water content of HVFA-cement-based materials were very limited in the literature, so the applicability and accuracy of Equations (13)–(15) cannot be verified in this study. Hence, further experimental studies will be performed to verify the applicability and accuracy of Equations (13)–(15). The basic properties (e.g., chemical composition, particle size distribution, hydraulic or pozzolanic reactivity) of raw materials are the key points that influence the hydration kinetics and strength development of cement-based materials. Therefore, the effect of the properties of raw materials will be the main concern when predicting the compressive strength of HVFA-cement-based materials.

## 4. Conclusions

This paper studied the hydration process and compressive strength development of HVFA-cement-based materials with different FA contents and *w/b*, and proposed a formula to predict the chemically bonded water content of HVFA-cement paste with *w/b*, FA content, and the curing age. Moreover, the relationship between the compressive strength and chemically bonded water content was established. According to the findings, the following conclusions can be drawn.

1. The compressive strength development of HVFA-cement mortar was rapid at early ages and tended to be slow at late ages. However, with the increase of FA content, the compressive strength grew faster at late ages. With the increase of *w/b* and FA content, the compressive strength of HVFA-cement mortar decreased. With the decrease of *w/b*, the effect of FA content on the compressive strength of mortar was more significant.
2. The chemically bound water content of HVFA-cement paste increased rapidly at early ages, and became slowly at late ages. The incorporation of FA reduced the chemically bound water content in HVFA-cement pastes. With the increase of *w/b*, the chemically bound water content increased, but *w/b* showed less influence at early ages and more significant influence at late ages.
3. The quantitative prediction formula for chemically bound water content was established based on *w/b*, FA content, and curing age. The equation could accurately predict the chemically bound water content of HVFA-cement-based materials.
4. There is a linear relationship between the chemically bound water content and compressive strength of HVFA-cement mortar. The compressive strength of HVFA-cement mortars could be predicted based on the chemically bound water content, within the scope of this study.

Since the compressive strength of FA concrete is closely related to the strength of FA mortar, the results of this study are helpful to develop the mix design method for HVFA concrete.

## 5. Limitations and Further Research Needs

Due to the lack of related experimental results, the applicability and accuracy of the proposed relationship between the chemically bound water content and compressive strength has not been verified in this study. Further studies will be carried out to verify the proposed relationship, and to consider the effect of raw materials’ properties.

## Figures and Tables

**Table 1:** Main chemical compositions of cement and FA/%.

Material	SiO_2_	Al_2_O_3_	Fe_2_O_3_	CaO	MgO	SO_3_	Loss
Cement	22.19	4.48	3.23	63.10	2.41	2.57	2.03
FA	47.12	36.92	3.97	6.79	0.93	0.69	1.99

**Table 2:** Mix proportions of HVFA-cement mortars.

Sample	Mix Proportions/g				
	Cement	FA	Sand	Water	Superplasticizer
FA0-0.4/0.5/0.6	450	0	1350	180/225/270	2.0/0.3/0
FA30-0.4/0.5/0.6	315	135	1350	180/225/270	2.0/0.3/0
FA40-0.4/0.5/0.6	270	180	1350	180/225/270	2.0/0.3/0
FA50-0.4/0.5/0.6	225	225	1350	180/225/270	2.0/0.3/0
FA60-0.4/0.5/0.6	180	270	1350	180/225/270	2.0/0.3/0
FA70-0.4/0.5/0.6	135	315	1350	180/225/270	2.0/0.3/0

Notes: 0.4/0.5/0.6 represents the water-to-binder ratios of 0.4, 0.5 and 0.6; 180/225/270 and 2.0/0.3/0 represent the water and superplasticizer dosages under the corresponding water-to-binder ratios, respectively.

**Table 3:** Fitting results on the relationship between the compressive strength and the chemically bound water content of HVFA-cement-based materials at different *w/b*.

w/b	Fitted Equation	R^2^	x-Axis Intersection
0.4	σ = 4.38 *w*_*cbw*_-12.70	0.97	2.90%
0.5	σ = 3.41 *w*_*cbw*_-12.00	0.94	3.52%
0.6	σ = 2.56 *w*_*cbw*_-10.52	0.95	4.11%

## Data Availability

The data that support the findings of this study are available from the corresponding author upon reasonable request.
